# Utilization of antenatal care services and associated factors among urban refugee adolescents in Kampala, Uganda

**DOI:** 10.4314/ahs.v25i3.10

**Published:** 2025-09

**Authors:** Olivia Nakisita, Christopher Garimoi Orach, Joseph Kagayi, Justine Bukenya, Arthur Bagonza, Elizabeth Nabiwemba, Christine Nalwadda

**Affiliations:** 1 Department of Community Health and Behavioral Sciences, Makerere University School of Public Health; 2 Department of Epidemiology and Biostatistics, Makerere University School of Public Health

**Keywords:** Maternal Health Services, Antenatal Care, Utilization, Adolescents, Urban Refugees, Kampala City

## Abstract

**Introduction:**

Uganda hosts an estimated 1.5 million refugees and 12.5% are urban refugees living in Kampala. Uganda is implementing an integrated healthcare system in which refugees and host populations utilize the same health care services.

**Objective:**

To assess the level of utilization of antenatal care services and associated factors among urban refugee adolescents in Kampala, Uganda.

**Methods:**

We conducted a cross-sectional study among 637 urban refugees who were adolescent mothers aged 10-19 years. Participants were interviewed using ODK software. The data were analyzed using Stata version 17. Uni-variate, bi-variate and multivariate analyses were performed, and the results reported as proportions, p values, odds ratios and confidence intervals.

**Results:**

Most (92.6%) had attended at least one ANC visit, however only a few (11.5%) attended the recommended WHO 8 visits. Less than half (41%) attended the initial ANC visit within the first trimester. The factors associated with ANC utilization included being married and living in an extended family. The factors associated with ANC attendance in the first trimester included being married, education and duration of stay in the area.

**Conclusions:**

Utilization of ANC by urban refugee adolescents in Kampala is sub-optimal. There is a need for targeted interventions to encourage adolescent refugees to utilize antenatal care services optimally.

## Background

Globally, there are 35.3 million refugees in 2024^1^. Over a quarter (26%) of the global population of refugees are found in sub-Saharan Africa^2^. Uganda is the third largest refugee-hosting country in the world and the first in Africa. Uganda hosts more than 1.5 million refugees, mainly from Sudan, South Sudan, Congo, Burundi, Rwanda, Somalia, Ethiopia and Eritrea^3^. Uganda has two types of refugees, those living in settlements as “settlement refugees” and those who register as “urban refugees”. Approximately 12.5% of the refugees in Uganda are urban refugees, and they survive like any other urban dweller and are free to use health services offered by the government^3^.

Uganda implements the Health Sector Integrated Refugee Response Plan, where both the refugees and host populations utilize the health services available without discrimination^5^. However urban refugee adolescents in Kampala face several challenges in accessing and utilizing maternal health services^6^, that include difficulty reaching the facility, lack of financial support, discrimination, disrespect by health workers and lack of privacy^7^.

Complications from pregnancy and childbirth are the leading causes of death among adolescent girls^8^, yet, adolescents have poorer maternal health care than older women^9^ and are less likely to utilize antenatal or delivery care services^10^ because they often perceive health care services as not easily accessible and therefore fail to utilize the services^11^.

All women, including refugees, have a right to good quality maternal health services^12^, however studies reveal low utilization of antenatal care services among migrant women in urban areas despite several initiatives to create demand and utilization for SRHR services for refugees^13^,^14^. Specific data on the level of utilization of antenatal care services by urban refugee adolescents in Uganda are not readily available. This study aimed to generate evidence on the level of utilization of antenatal care services and associated factors among urban refugee adolescents in Uganda.

## Methods

We conducted a cross-sectional study between November and December 2023 in Kampala city, Uganda, quantitative methods of data collection were used. We conducted interviews among 641 registered urban refugees who were pregnant 10-19, five years prior to the study, regardless of pregnancy outcome. The study participants were refugees from Congo, Burundi, Rwanda, Ethiopia, Sudan, South Sudan, Somalia, Eritrea, and Tanzania. They were identified from the Central, Rubaga, Makindye, Kawempe, and Nakawa divisions. The sample size was determined by the formula of random sampling using single proportions given by Kish Leslie, 1965.

Deff = design effect, a measure of efficiency of cluster sampling compared to a direct simple random sample taken as 1.5 from similar studies;
Z = standard normal value corresponding to the 95% confidence interval (1.96),
P = the anticipated population proportion of the study outcome (this proportion was not known, and we conservatively used 50%), d = margin of allowable error (5%) NR = expected non-response assumed to be 10% (0.1) based on similar studies. The sample size was estimated to be 641.

### Data collection procedures

The data were collected through face-to-face interviews using a semi structured questionnaire administered through mobile phones using Open Data Kit (ODK) software. Three parishes were randomly sampled from each division, while 3 villages were randomly selected from each parish and included in the study. The respondents were systematically sampled where every fifth household was considered. Only one eligible respondent was selected per household. If no eligible respondent was found, the next household was considered. To ensure quality of the data, ten research assistants were selected based on their technical ability and trained for two days. The tool was pretested with a similar group of people in the parishes that were not selected to participate in the study. Thereafter, corrections and adjustments were made to the tool.

The utilization of antenatal care services was a normal measurement categorized as a binary variable where (Yes) meant that the service was utilized and (No) meant that the service was not utilized. Optimal utilization of ANC was defined as (i) attending 8 visits based on WHO guidelines and (ii) attending the initial ANC visit within the first 12 weeks of pregnancy.

### Data Management and Analysis

The data were collected in ODK and exported to Excel for cleaning. Analysis was performed with STATA version 17. Univariate analysis was conducted, and the frequencies of each study variable were generated. Bivariate analysis using the chi-square test was performed to test for associations between the dependent and independent variables, while multivariate analysis using logistic regression was conducted to determine the predictors of ANC attendance. The results are presented in tabular form as proportions, p values, odds ratios and confidence intervals.

### Ethical Considerations

Ethical approval was obtained from the Makerere University School of Public Research Ethics Committee (Ref: SPH-2023-449). Clearance was received from the Uganda National Council of Science and Technology (Ref: HS3193ES). Informed written consent was obtained and confidentiality was ensured by conducting interviews in private, anonymizing respondents and storing data on a password-protected computer which was only accessed by authorized research team members.

## Results

Social Demographic Characteristics of the Respondents We achieved a response rate of 637/641 (99.4%). The majority 600/637 (94.2%) of the respondents reported becoming pregnant between the ages of 15 and 19 years, and 37/637 (5.8%) between 10 and 14 years, with the youngest being 12 years. The mean age was 17.1 (SD ±1.9) years, and the median age was 18 years (IQR 16-19). About 206/637 (32.3%) of the respondents were married, 137/206 (66.5%) were in polygamous marriages, and over half 357/637 (56%) lived in extended families. A good number (69.6%) of the adolescent mothers were unemployed, and 376/637 (60%) had low levels of education (Table 1).

**Table 1:** Socio-demographic characteristics of the respondents

Variable	Frequency N=637	Percentage (%)
Age at first pregnancy 10-14 years	37	94.2
15-19 years	600	5.8
		
Age at first pregnancy (in years) (Median (IQR)	18 (IQR 16-19)	
		
Mean Age	17.1 (SD 1.9)	
		
**Marital status**		
Married	206	32.3
Single	281	44.1
Widowed	12	1.9
Divorced/separated	24	3.8
No response	114	17.9
		
**Type of Marriage**		
Monogamous	69	33.5
Polygamous	137	66.5
		
**Family Type**		
Extended	357	56.0
Nuclear	280	44.0
		
**Occupation**		
Subsistence farmer	15	2.3
Full time house wife	124	19.5
None	319	50.1
Trader	147	23.1
Other	32	5.0
		
**Education level**		
No Formal Education	134	21.0
Primary	242	38.0
Secondary	186	29.2
Tertiary	59	9.3
University	16	2.5
		
**Religion**		
Protestant	147	23.1
Catholic	136	21.4
Muslim	250	39.2
Born Again	9	15.2
Other	7	1.1
		
**Period Living in Kampala**		
0 – 2 years	62	9.7
3 – 5 years	311	48.8
Over 5 years	264	41.5
		
**Spousal/partner Occupation**		
No spouse	281	44.1
Unemployed	183	28.7
Skilled employment	51	8.0
Subsistence farming	12	1.9
Trader	85	13.4
Other	25	3.9
		
**Spousal/Partner Education level**		
No spouse	281	44.1
None	141	22.1
Primary	62	9.7
Secondary	88	13.8
Tertiary	39	6.2
University	26	4.1

Many of the respondents were Muslims 250/637 (39.2%), Protestants 147/637 (23.1%) and Catholics 136/637 (21.4%). Almost half 311/637 (48.8%) had lived in Kampala for three to five years, while 264/637 (40%) had lived in Kampala for more than 5 years (Table 1).

### Utilization of Antenatal Care Services

This study revealed that 590 (92.6%) of the participants attended antenatal care (ANC) services at least once. The majority, 428/590 (72.5%), attended antenatal care four or more times during pregnancy, and only a few, 68/590 (11.5%), attended the WHO 8 recommended visits during pregnancy. The average antenatal care attendance of the participants was five (5). Less than half 245/590 (41.5%) of the participants attended antenatal care within the first three months of pregnancy. The majority of the participants 517/590 (87.6%) attended antenatal care services from government health facilities (Table 2).

**Table 2:** Utilization of Antenatal Care Services by Urban Refugees Adolescent in Kampala City

Variable	Frequency N=637	Percentage (%)
**Did you attend antenatal care services**		
Yes	590	92.6
No	47	7.4
		
**How many times did you attend ANC**		
1-3 times	162	27.5
4 and more times	428	72.5
**At what age of the pregnancy did you attend your first ANC**		
0-3 months	245	41.5
4-6 months	291	49.3
7-9 months	54	9.2
		
**Where did you attend ANC**		
Government Health Facility	517	87.6
Private Facility	49	8.3
Private non for profit Facility	24	4.1
		
**How did you get information about these ANC services**		
Friends and Family	235	39.8
Health Workers	355	60.2
		
**How far was the health facility from your home**		
Very Far	28	4.7
Far	127	21.5
Not very near	247	41.9
Near	188	31.9
		
**What means of transport did you use to go for ANC**		
Boda Boda	183	31.0
Walked there	187	31.7
Taxi	112	19.0
Private Vehicle	48	8.2
Multiple means	60	10.1
		
**How do you consider the waiting time at the ANC service poin**t		
Appropriate	336	56.9
Too long	254	43.1
		
**How do you consider the transport cost to get to the facility**		
		
Affordable	190	32.2
Expensive	268	45.4
Fair	132	22.4
		
**Did you get all the information you needed from the health workers during ANC**		
		
No	61	10.3
Yes	529	89.7

### Factors associated with the utilization of ANC services by adolescent urban refugees

The study findings show that factors significantly associated with the use of ANC services include marital status (p value of 0.000), family type (p value of 0.001), occupation (p value of 0.000), and nature of the facility (p value of 0.000). (Table 3)

**Table 3:** Bivariable analysis of the number of ANC visits using a chi-square test

Variable	X^2^ Statistic Degree of Freedom	P Value (0.05)
**Age at first pregnancy (years)**		
10 – 14	5.0 (2)	0.025*
15 – 19		
**Marital status**		
Married		
Single		
Widowed		
Divorced/separated	33.7 (8)	0.000*
No response		
**Family Type**		
Extended	13.9(2)	0.001*
Nuclear		
**Occupation**		
Subsistence farmer		
Full time house wife	28.7 (8)	0.000*
None		
Trader		
Other Specify		
		
**Education level**		
None	13.7 (8)	0.090
Primary		
Secondary		
Tertiary		
University		
**Religion**		
Protestant		
Catholic	13.8(8)	0.085
Muslim		
Born Again		
Other		
**Period Living in Kampala**		
0 – 2 years	10.6(2)	0.005*
3 – 5 years		
5 years and above		
**Distance to the facility**		
Near		
Not very near	4.75(6)	0.576
Not very far		
Far		
**Nature of the facility**		
Government Facility	26.5(4)	0.000*
Private Non for Profit		
Private Facility		

* Statistically significant p values

### Multiple variable analysis of factors associated with ANC attendance

The study revealed that the factors associated with ANC attendance were being married [OR: 3.22 (1.371, 7.56)] and living in an extended family [OR: 2.093 (1.08, 4.054)].

### Factors associated with ANC attendance in the first trimester

The study revealed that the factors associated with ANC attendance in the first trimester were being married [OR: 2.97 (1.962, 4.503)], education [2.01 (0.744, 5.39] and duration of residence at the location [2.53 (1.685, 3.801)].

## Discussion

### Utilization of ANC services among adolescent urban refugees in Kampala

The majority (92.6%) of the urban refugee adolescents in Kampala attended at least one ANC visit. This finding is similar to the findings of the 2016 UDHS study in Uganda, where 95% of the general population of women of reproductive age attended ANC care at least once^15^. This finding could be because Kampala city, has a good network of government, PNFP and private health facilities that can easily be accessed geographically, financially and temporally.

This study revealed that the majority (72.5%) of the urban refugee adolescents in Kampala attended four or more ANC visits. This finding is similar to the findings of the general population, where the Uganda Demographic Health Survey of 2016 revealed that 72% of those with ANC attendance four or more times^15^. This shows that the health system is integrated and does not discriminate against refugees in regard to health service delivery.

Furthermore, the study revealed that, on average, the study participants attended 5 ANC visits. This is almost similar to a study of Syrian refugees in Lebanon that revealed an average of 6 antenatal care visits^16^. This is still lower than the recommended WHO 8 ANC visits which is an indication that more interventions are needed to increase the number times adolescents refugees attend ANC to improve their pregnancy outcomes.

The study revealed that less than half of the adolescents (41%) attended their first antenatal care visit within the first trimester. Our finding is similar to that of a study carried out in Rwanda that revealed that 46% of adolescent mothers attended ANC in the first trimester^17^. Our study findings concur with a study carried out in the United States that revealed that more refugees and immigrants have their first obstetric visits after 12 weeks of gestation and have fewer prenatal visits than the host population^18^. This could be because adolescents face stigma and discrimination when they become pregnant and therefore fear disclosing their pregnancy status early.

### Factors Associated with ANC Attendance

Findings show that being married issignificantly associated with ANC attendance. This study concurs with a study carried out in Indonesia that concluded that married women have a better chance of making complete ANC visits than single women^19^. This implies that being married increases the chances of an adolescent attending the recommended 8 ANC visits and yet adolescents are too young to be married which reduces their support system to attend ANC.

This study showed that living in an extended family increases the chances of one attending the recommended 8 ANC visits. This could be because adolescents who become pregnant are often discriminated against, isolated, labeled and victimized by their parents because of the shame that teenage pregnancy brings to their families. Therefore, an extended family member is likely to support pregnant adolescents, as they need support from their families and friends to be able to attend ANC optimally.

This study revealed a statistically significant association between education level and ANC in the first trimester. This may be explained by the fact that education increases the chances of accessing information and income, which enhances decision making to utilize ANC services. Similarly, a study carried out in Ethiopia revealed that the level of education was significantly associated with the early initiation of ANC^20^. A systematic review conducted in conflict situations also concluded that education is one of the factors impacting antenatal care utilization^21^. Furthermore, a study carried out among Somali refugees underscored literacy as playing and determining whether a mother will utilize maternal health services^22^.

This study found a significant association between attending ANC in the first trimester and marriage status. Our findings concur with a study carried out in Indonesia that concluded that married women have a better chance of making complete ANC^19^. This could be because the girls who marry get support from their spouses in attending ANC visits and that those who are not married are at a disadvantage.

The duration of one's residence at the location was significantly associated with attending ANC in the first trimester. This can be explained by the fact that the longer one lives in an area, the more one learns about the availability of existing health services. This enhances their chances of utilizing maternal health care services, including ANC.

## Conclusion and Recommendations

The utilization of antenatal care by urban refugee adolescents is sub optimal, with only one in ten attending the 8 visits recommended by the WHO and four in ten attending their initial ANC visit within the first 12 weeks of pregnancy. This implies that the refugee adolescents may experience pregnancy complications that put them and their babies at a risk of maternal and neonatal deaths. There is a need for targeted interventions, including creating awareness through telephone reminders and home visits, to encourage urban refugee adolescents to utilize antenatal care services optimally with 8 visits and ANC in the first trimester.

## Strengths and Limitations of the study

The strength of this study is that the findings are generalizable to urban refugee adolescents in Kampala. This study can be easily replicated in other settings.

The limitations of this study is that this was a quantitative study and there is need for a qualitative study to explore why the urban refugee adolescents don't attend ANC timely and adequately.

## Figures and Tables

**Table 4:** Factors associated with ANC attendance

Determinants	Un adjusted OR	Adjusted OR

	OR (95% CI)	OR (95% CI)
**Age group**		
Below 15 yrs	Ref (OR = 1.000)	Ref (OR = 1.000)
15-19 yrs	0.320(0.076, 1.346)	0.291(0.065, 1.304)
**Duration resident at location**		
1-4 yrs	Ref (OR=1.000)	Ref (OR=1.000)
5+ yrs	1.888(1.0401, 3.428)	1.718(0.881, 3.351)
		
**Marital status**		
Single	Ref (OR=1.000)	Ref (OR=1.000)
Married	3.521(1.597, 7.763)	3.22(1.371, 7.56)*
Divorced/Separated	1.000(0.282, 3.513)	1.308(0.322, 5.307)
Widowed	1.565(0.196, 12.496)	2.114(0.244, 18.286)
		
**Family type**		
Nuclear	Ref (OR=1.000)	Ref (OR=1.000)
Extended	1.493(0.823, 2.708)	2.093(1.08, 4.054)*
**Education**		
None	Ref (OR=1.000)	Ref (OR = 1.000)
Primary	0.269(0.780, 0.926)	1.294(0.306, 5.461)
Secondary	0.227(0.065, 0.793)	1.194(0.277, 5.149)
Univ/Tertiary	0.192(0.0491, 0.747)	0.724(0.151, 3.478)
**Religion**		
Catholic	Ref (OR=1.000)	Ref (OR=1.000)
Protestant	1.379(0.528, 3.603)	1.132(0.423, 3.027)
Born Again	0.434(0.186, 1.012)	0.446(0.187, 1.066)
Moslem	1.724(0.713, 4.170)	0.517(0.199, 1.346)
Others	0.106(0.021, 0.540)	0.061(0.01, 0.384)

* statistically significant p values

**Table 5:** Determinants of ANC attendance in the first trimester

Determinants	Un adjusted OR	Adjusted OR

	OR (95% CI)	OR (95% CI)
**Age group**		
Below 15 yrs	Ref (OR = 1.000)	Ref (OR = 1.000)
15-19 yrs	0.851(0.520,1.391)	1.461(0.8,2.666)
**Duration residence at location**		
1-4 yrs	Ref (OR=1.000)	Ref (OR=1.000)
5+ yrs	1.912(1.355,2.700)	2.531(1.685,3.801)*
		
**Marital status**		
Single	Ref (OR=1.000)	Ref (OR=1.000)
Married	3.035(2.090,4.408)	2.972(1.962,4.503)*
Divorced/Separated	1.156(0.488,2.739)	0.938(0.376,2.34)
Widowed	2.700(0.834,8.726)	1.872(0.556,6.305)
		
**Family type**		
Nuclear	Ref (OR = 1.000)	Ref (OR = 1.000)
Extended	2.088(1.498,2.909)	1.101 (0.732,1.655)
**Education**		
None	Ref (OR=1.000)	Ref (OR=1.000)
Primary	7.014(3.75,13.119)	2.062(0.774,5.492)
Secondary	8.726(4.6,16.553)	2.005(0.744,5.399)*
Univ/Tertiary	9.559(4.609,19.826)	1.936(0.672,5.575)
		
**Religion**		
Catholic	Ref (OR=1.000)	Ref (OR=1.000)
Protestant	0.803(0.499,1.292)	0.716(0.427,1.201)
Born Again	1.083(0.641,1.83)	1.127(0.642,1.98)
Moslem	0.649(0.422,0.996)	0.977(0.565,1.688)
Others	0.979(0.211,4.542)	0.756(0.127,4.515)

* Statistically significant p values
